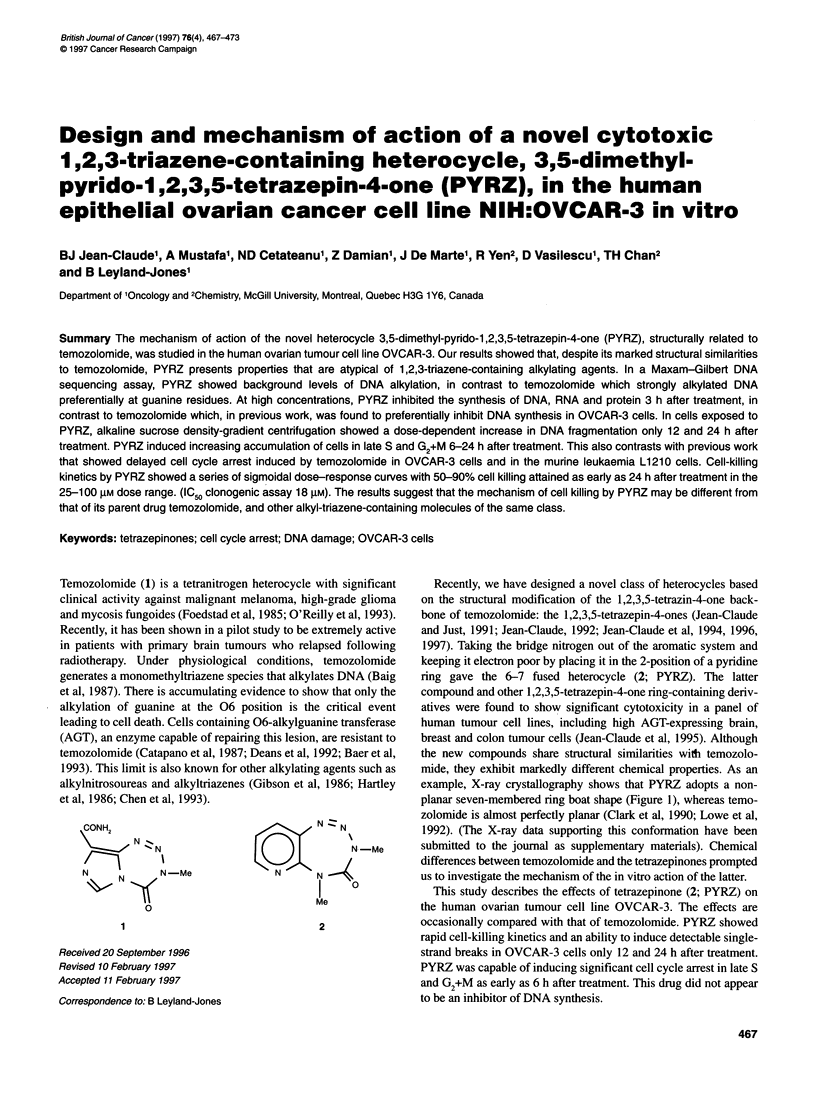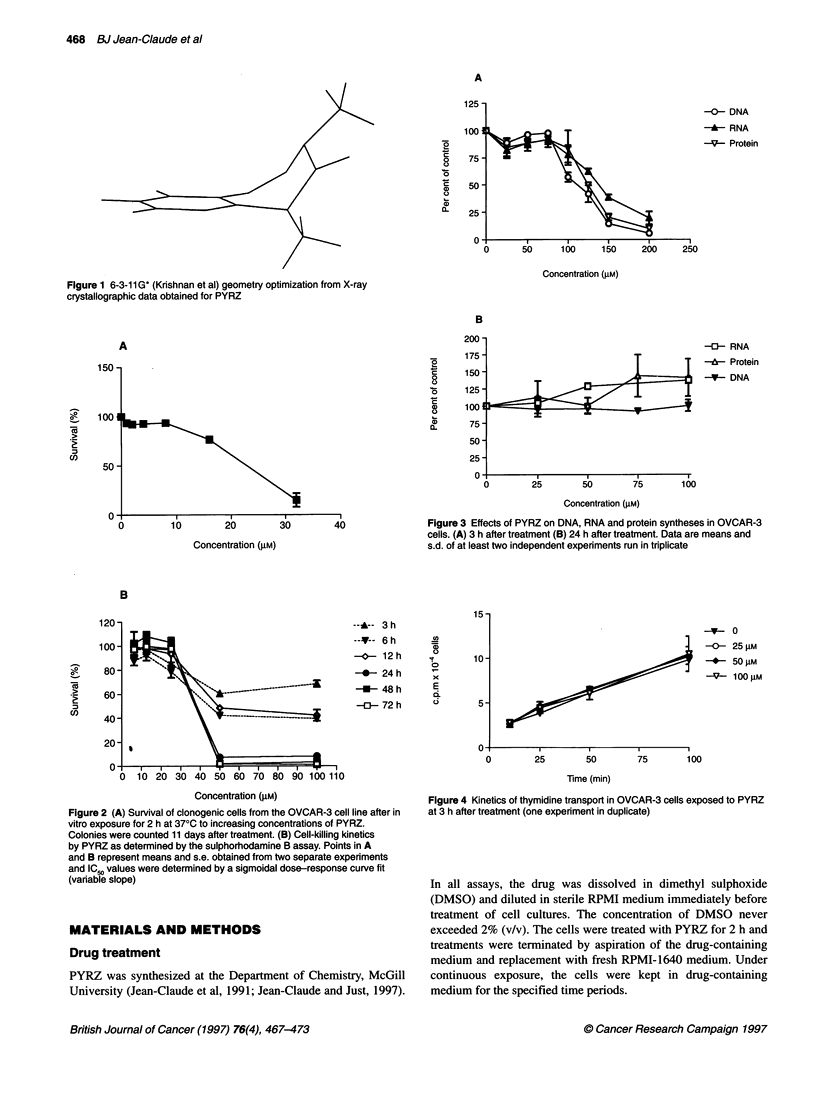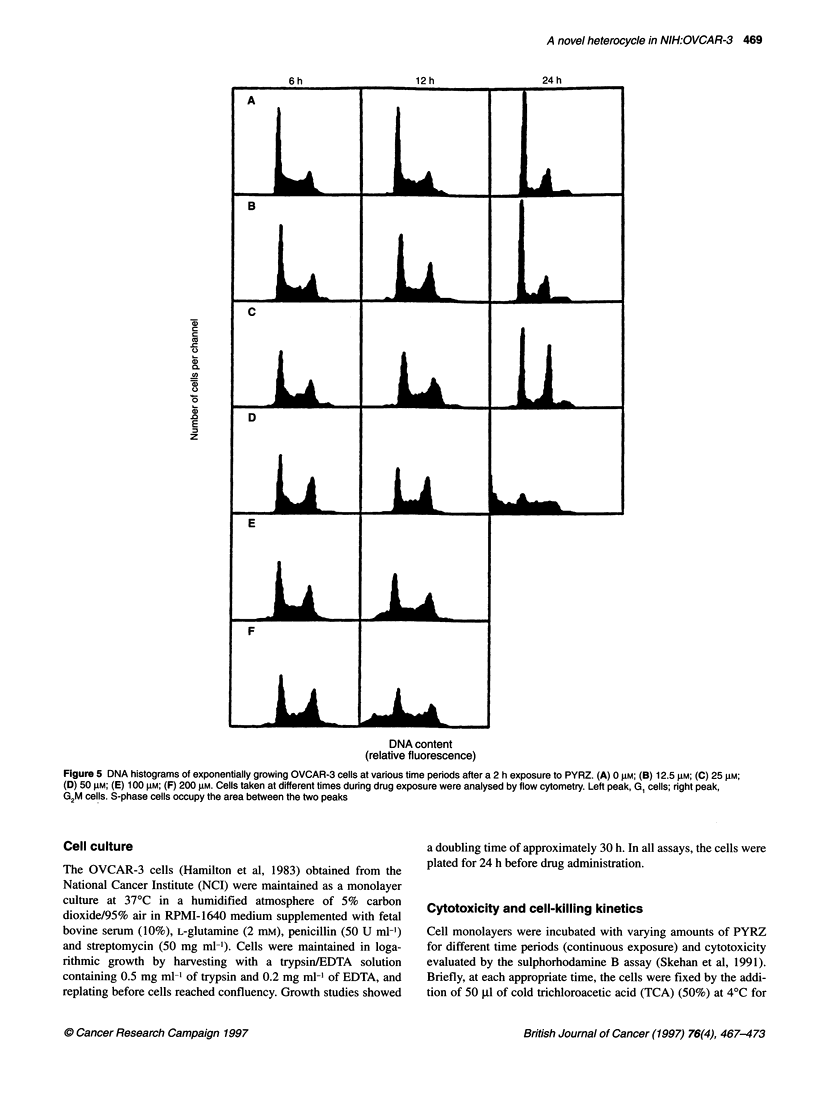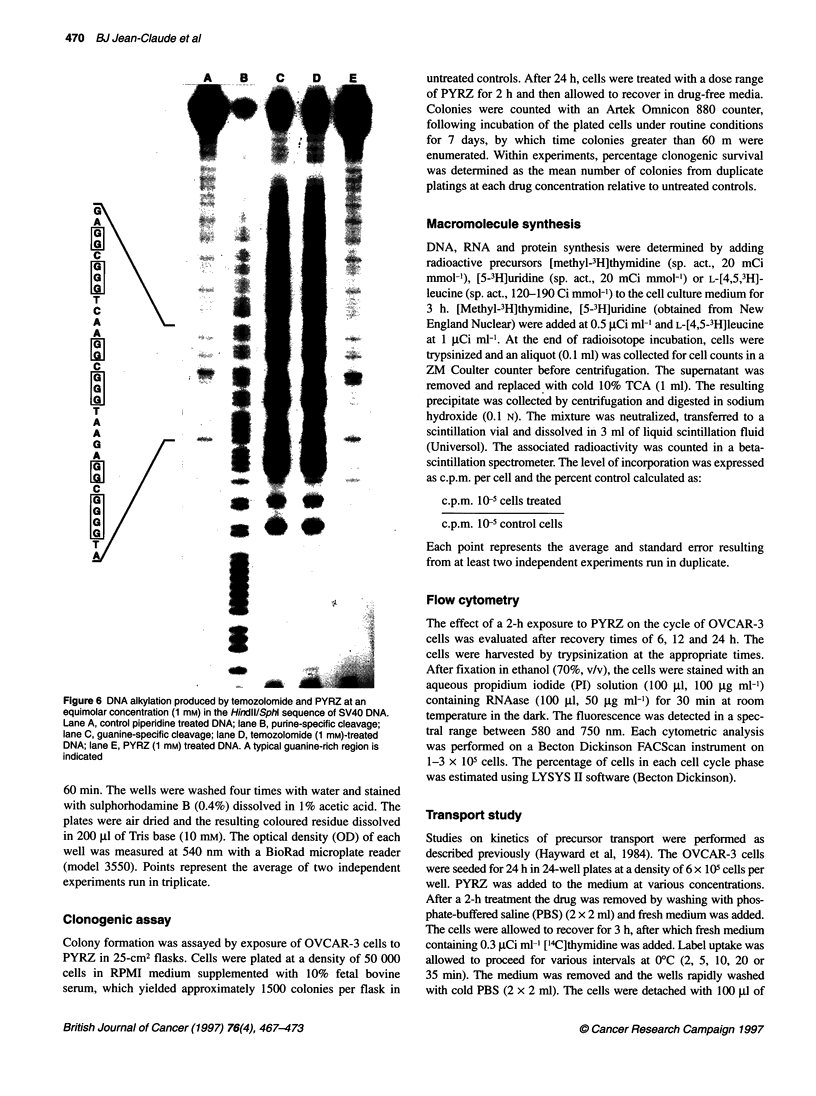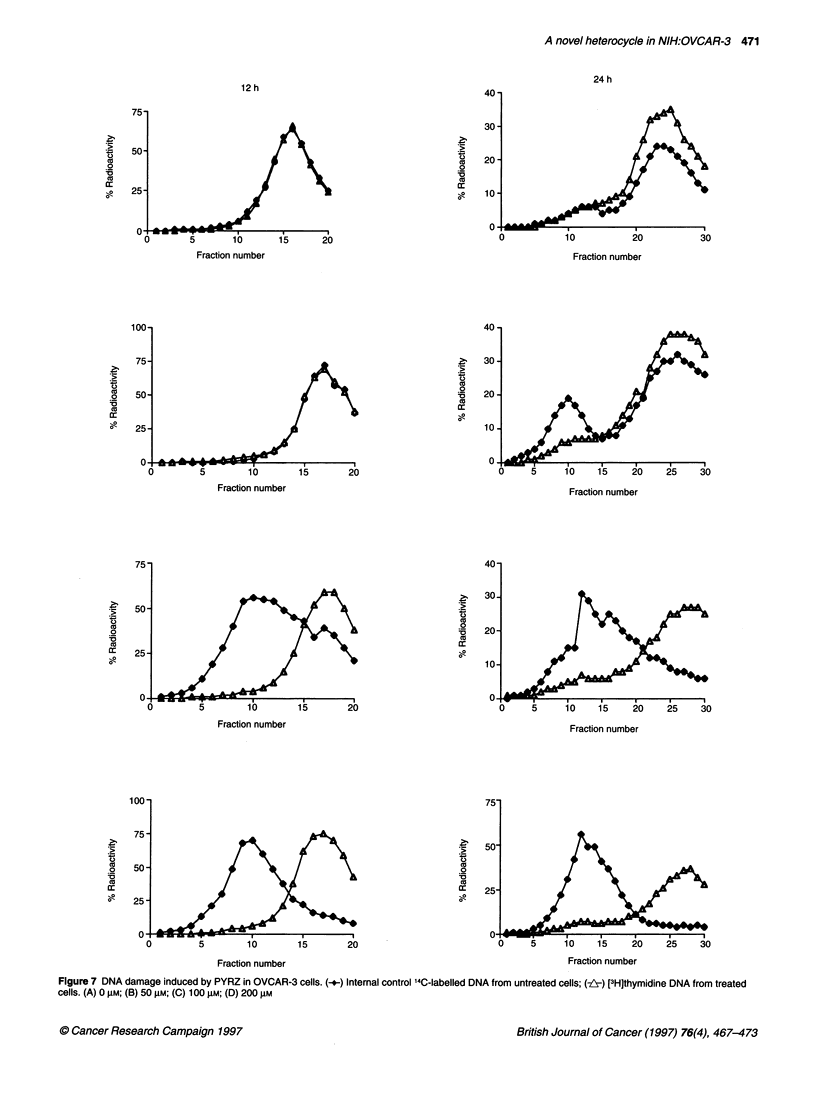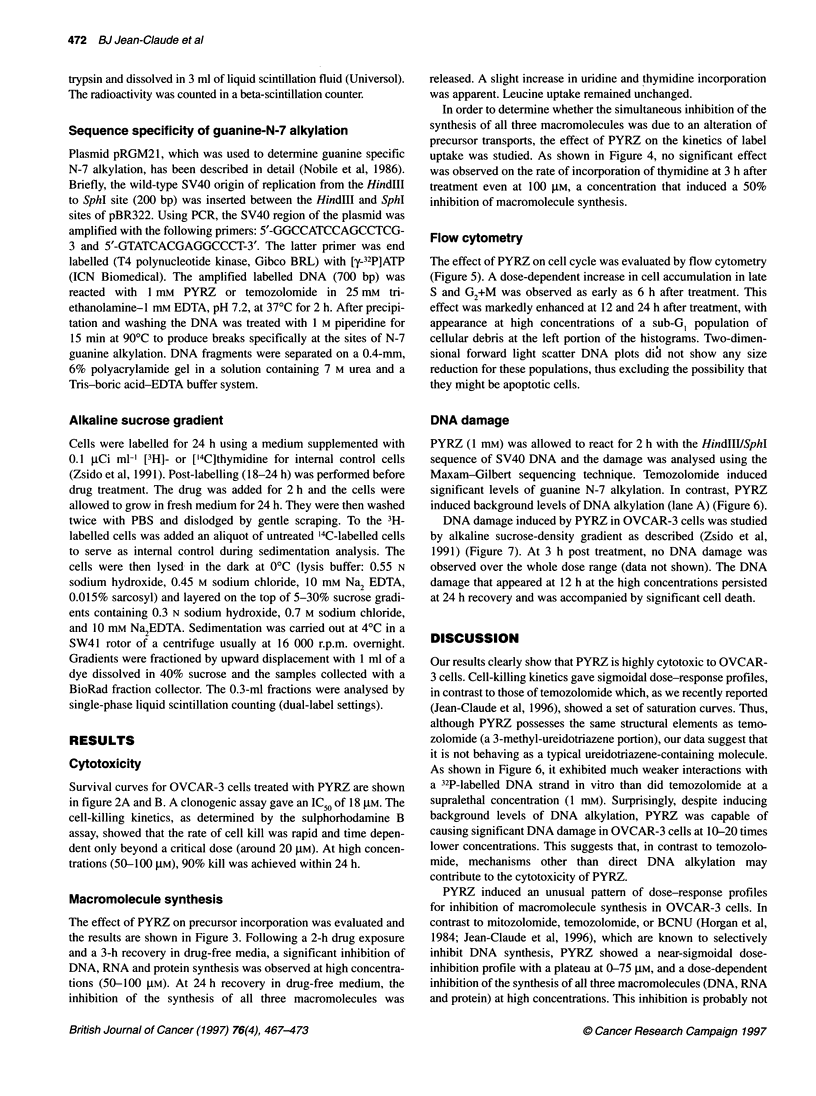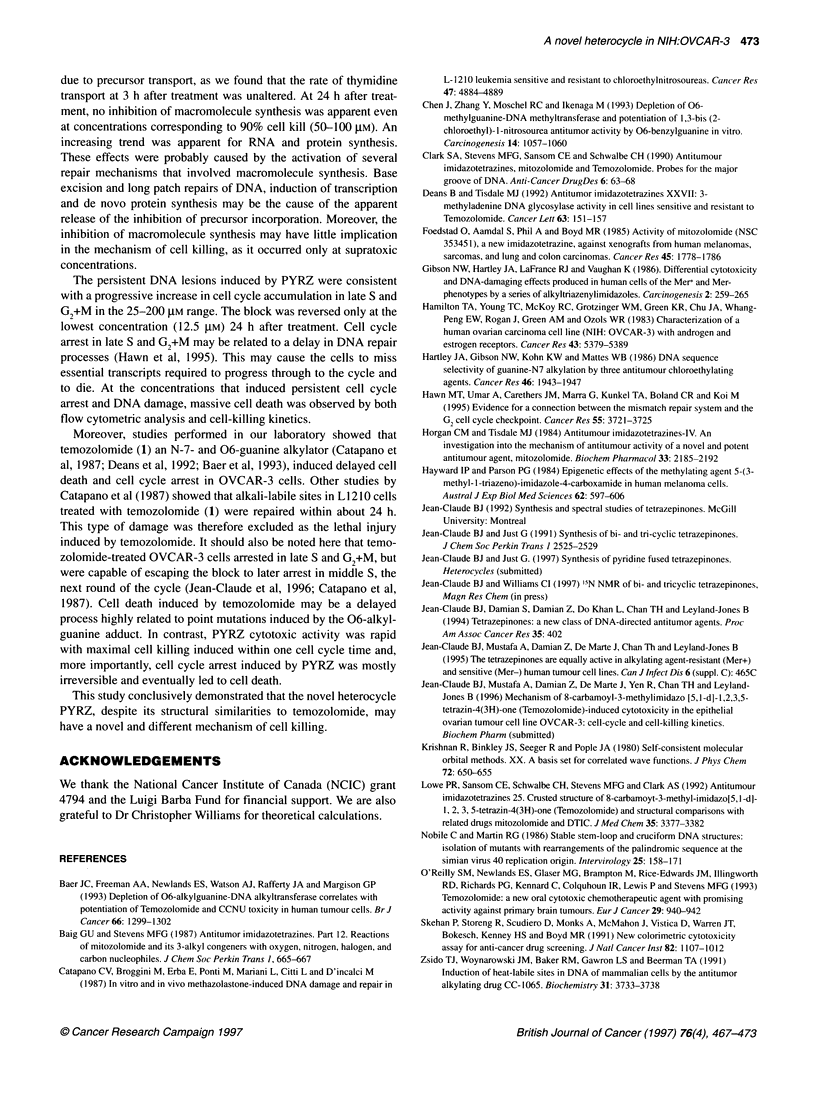# Design and mechanism of action of a novel cytotoxic 1,2,3-triazene-containing heterocycle, 3,5-dimethyl-pyrido-1,2,3,5-tetrazepin-4-one (PYRZ), in the human epithelial ovarian cancer cell line NIH:OVCAR-3 in vitro.

**DOI:** 10.1038/bjc.1997.411

**Published:** 1997

**Authors:** B. J. Jean-Claude, A. Mustafa, N. D. Cetateanu, Z. Damian, J. De Marte, R. Yen, D. Vasilescu, T. H. Chan, B. Leyland-Jones

**Affiliations:** Department of Oncology, McGill University, Montreal, Quebec, Canada.

## Abstract

**Images:**


British Journal of Cancer (1997) 76(4), 467–473
© 1997 Cancer Research Campaign

### Design and mechanism of action of a novel cytotoxic 1,2,3-triazene-containing heterocycle, 3,5-dimethyl-pyrido-1,2,3,5-tetrazepin-4-one (PYRZ), in the human epithelial ovarian cancer cell line NIH:OVCAR-3 in vitro

**BJ Jean-Claude[1], A Mustafa[1], ND Cetateanu[1], Z Damian[1], J De Marte[1], R Yen[2], D Vasilescu[1], TH Chan[2] and B Leyland-Jones[1]**

Department of [1]Oncology and [2]Chemistry, McGill University, Montreal, Quebec H3G 1Y6, Canada

**Summary** The mechanism of action of the novel heterocycle 3,5-dimethyl-pyrido-1,2,3,5-tetrazepin-4-one (PYRZ), structurally related to temozolomide, was studied in the human ovarian tumour cell line OVCAR-3. Our results showed that, despite its marked structural similarities to temozolomide, PYRZ presents properties that are atypical of 1,2,3-triazene-containing alkylating agents. In a Maxam–Gilbert DNA sequencing assay, PYRZ showed background levels of DNA alkylation, in contrast to temozolomide which strongly alkylated DNA preferentially at guanine residues. At high concentrations, PYRZ inhibited the synthesis of DNA, RNA and protein 3 h after treatment, in contrast to temozolomide which, in previous work, was found to preferentially inhibit DNA synthesis in OVCAR-3 cells. In cells exposed to PYRZ, alkaline sucrose density-gradient centrifugation showed a dose-dependent increase in DNA fragmentation only 12 and 24 h after treatment. PYRZ induced increasing accumulation of cells in late S and $G_2$+M 6–24 h after treatment. This also contrasts with previous work that showed delayed cell cycle arrest induced by temozolomide in OVCAR-3 cells and in the murine leukaemia L1210 cells. Cell-killing kinetics by PYRZ showed a series of sigmoidal dose–response curves with 50–90% cell killing attained as early as 24 h after treatment in the 25–100 μM dose range. ($IC_{50}$ clonogenic assay 18 μM). The results suggest that the mechanism of cell killing by PYRZ may be different from that of its parent drug temozolomide, and other alkyl-triazene-containing molecules of the same class.

**Keywords:** tetrazepinones; cell cycle arrest; DNA damage; OVCAR-3 cells

Temozolomide (**1**) is a tetranitrogen heterocycle with significant clinical activity against malignant melanoma, high-grade glioma and mycosis fungoides (Foedstad et al, 1985; O'Reilly et al, 1993). Recently, it has been shown in a pilot study to be extremely active in patients with primary brain tumours who relapsed following radiotherapy. Under physiological conditions, temozolomide generates a monomethyltriazene species that alkylates DNA (Baig et al, 1987). There is accumulating evidence to show that only the alkylation of guanine at the O6 position is the critical event leading to cell death. Cells containing O6-alkylguanine transferase (AGT), an enzyme capable of repairing this lesion, are resistant to temozolomide (Catapano et al, 1987; Deans et al, 1992; Baer et al, 1993). This limit is also known for other alkylating agents such as alkylnitrosoureas and alkyltriazenes (Gibson et al, 1986; Hartley et al, 1986; Chen et al, 1993).

**1**

**2**

*Received 20 September 1996*
*Revised 10 February 1997*
*Accepted 11 February 1997*

*Correspondence to:* B Leyland-Jones

Recently, we have designed a novel class of heterocycles based on the structural modification of the 1,2,3,5-tetrazin-4-one backbone of temozolomide: the 1,2,3,5-tetrazepin-4-ones (Jean-Claude and Just, 1991; Jean-Claude, 1992; Jean-Claude et al, 1994, 1996, 1997). Taking the bridge nitrogen out of the aromatic system and keeping it electron poor by placing it in the 2-position of a pyridine ring gave the 6–7 fused heterocycle (**2**; PYRZ). The latter compound and other 1,2,3,5-tetrazepin-4-one ring-containing derivatives were found to show significant cytotoxicity in a panel of human tumour cell lines, including high AGT-expressing brain, breast and colon tumour cells (Jean-Claude et al, 1995). Although the new compounds share structural similarities with temozolomide, they exhibit markedly different chemical properties. As an example, X-ray crystallography shows that PYRZ adopts a non-planar seven-membered ring boat shape (Figure 1), whereas temozolomide is almost perfectly planar (Clark et al, 1990; Lowe et al, 1992). (The X-ray data supporting this conformation have been submitted to the journal as supplementary materials). Chemical differences between temozolomide and the tetrazepinones prompted us to investigate the mechanism of the in vitro action of the latter.

This study describes the effects of tetrazepinone (**2**; PYRZ) on the human ovarian tumour cell line OVCAR-3. The effects are occasionally compared with that of temozolomide. PYRZ showed rapid cell-killing kinetics and an ability to induce detectable single-strand breaks in OVCAR-3 cells only 12 and 24 h after treatment. PYRZ was capable of inducing significant cell cycle arrest in late S and $G_2$+M as early as 6 h after treatment. This drug did not appear to be an inhibitor of DNA synthesis.

**Figure 1** 6-3-11G* (Krishnan et al) geometry optimization from X-ray crystallographic data obtained for PYRZ

**Figure 3** Effects of PYRZ on DNA, RNA and protein syntheses in OVCAR-3 cells. (**A**) 3 h after treatment (**B**) 24 h after treatment. Data are means and s.d. of at least two independent experiments run in triplicate

**Figure 2** (**A**) Survival of clonogenic cells from the OVCAR-3 cell line after in vitro exposure for 2 h at 37°C to increasing concentrations of PYRZ. Colonies were counted 11 days after treatment. (**B**) Cell-killing kinetics by PYRZ as determined by the sulphorhodamine B assay. Points in **A** and **B** represent means and s.e. obtained from two separate experiments and $IC_{50}$ values were determined by a sigmoidal dose–response curve fit (variable slope)

**Figure 4** Kinetics of thymidine transport in OVCAR-3 cells exposed to PYRZ at 3 h after treatment (one experiment in duplicate)

### MATERIALS AND METHODS

### Drug treatment

PYRZ was synthesized at the Department of Chemistry, McGill University (Jean-Claude et al, 1991; Jean-Claude and Just, 1997). In all assays, the drug was dissolved in dimethyl sulphoxide (DMSO) and diluted in sterile RPMI medium immediately before treatment of cell cultures. The concentration of DMSO never exceeded 2% (v/v). The cells were treated with PYRZ for 2 h and treatments were terminated by aspiration of the drug-containing medium and replacement with fresh RPMI-1640 medium. Under continuous exposure, the cells were kept in drug-containing medium for the specified time periods.

 © Cancer Research Campaign 1997

**Figure 5** DNA histograms of exponentially growing OVCAR-3 cells at various time periods after a 2 h exposure to PYRZ. (**A**) 0 µM; (**B**) 12.5 µM; (**C**) 25 µM; (**D**) 50 µM; (**E**) 100 µM; (**F**) 200 µM. Cells taken at different times during drug exposure were analysed by flow cytometry. Left peak, $G_1$ cells; right peak, $G_2M$ cells. S-phase cells occupy the area between the two peaks

### Cell culture

The OVCAR-3 cells (Hamilton et al, 1983) obtained from the National Cancer Institute (NCI) were maintained as a monolayer culture at 37°C in a humidified atmosphere of 5% carbon dioxide/95% air in RPMI-1640 medium supplemented with fetal bovine serum (10%), L-glutamine (2 mM), penicillin (50 U $ml^{-1}$) and streptomycin (50 mg $ml^{-1}$). Cells were maintained in logarithmic growth by harvesting with a trypsin/EDTA solution containing 0.5 mg $ml^{-1}$ of trypsin and 0.2 mg $ml^{-1}$ of EDTA, and replating before cells reached confluency. Growth studies showed a doubling time of approximately 30 h. In all assays, the cells were plated for 24 h before drug administration.

### Cytotoxicity and cell-killing kinetics

Cell monolayers were incubated with varying amounts of PYRZ for different time periods (continuous exposure) and cytotoxicity evaluated by the sulphorhodamine B assay (Skehan et al, 1991). Briefly, at each appropriate time, the cells were fixed by the addition of 50 µl of cold trichloroacetic acid (TCA) (50%) at 4°C for

© Cancer Research Campaign 1997

**Figure 6** DNA alkylation produced by temozolomide and PYRZ at an equimolar concentration (1 mM) in the *HindII/SphI* sequence of SV40 DNA. Lane A, control piperidine treated DNA; lane B, purine-specific cleavage; lane C, guanine-specific cleavage; lane D, temozolomide (1 mM)-treated DNA; lane E, PYRZ (1 mM) treated DNA. A typical guanine-rich region is indicated

60 min. The wells were washed four times with water and stained with sulphorhodamine B (0.4%) dissolved in 1% acetic acid. The plates were air dried and the resulting coloured residue dissolved in 200 µl of Tris base (10 mM). The optical density (OD) of each well was measured at 540 nm with a BioRad microplate reader (model 3550). Points represent the average of two independent experiments run in triplicate.

### Clonogenic assay

Colony formation was assayed by exposure of OVCAR-3 cells to PYRZ in 25-$cm^2$ flasks. Cells were plated at a density of 50 000 cells in RPMI medium supplemented with 10% fetal bovine serum, which yielded approximately 1500 colonies per flask in untreated controls. After 24 h, cells were treated with a dose range of PYRZ for 2 h and then allowed to recover in drug-free media. Colonies were counted with an Artek Omnicon 880 counter, following incubation of the plated cells under routine conditions for 7 days, by which time colonies greater than 60 m were enumerated. Within experiments, percentage clonogenic survival was determined as the mean number of colonies from duplicate platings at each drug concentration relative to untreated controls.

### Macromolecule synthesis

DNA, RNA and protein synthesis were determined by adding radioactive precursors [methyl-$^3$H]thymidine (sp. act., 20 mCi $mmol^{-1}$), [5-$^3$H]uridine (sp. act., 20 mCi $mmol^{-1}$) or L-[4,5,$^3$H]-leucine (sp. act., 120–190 Ci $mmol^{-1}$) to the cell culture medium for 3 h. [Methyl-$^3$H]thymidine, [5-$^3$H]uridine (obtained from New England Nuclear) were added at 0.5 µCi $ml^{-1}$ and L-[4,5-$^3$H]leucine at 1 µCi $ml^{-1}$. At the end of radioisotope incubation, cells were trypsinized and an aliquot (0.1 ml) was collected for cell counts in a ZM Coulter counter before centrifugation. The supernatant was removed and replaced with cold 10% TCA (1 ml). The resulting precipitate was collected by centrifugation and digested in sodium hydroxide (0.1 N). The mixture was neutralized, transferred to a scintillation vial and dissolved in 3 ml of liquid scintillation fluid (Universol). The associated radioactivity was counted in a beta-scintillation spectrometer. The level of incorporation was expressed as c.p.m. per cell and the percent control calculated as:

$$\frac{\text{c.p.m. } 10^{-5} \text{ cells treated}}{\text{c.p.m. } 10^{-5} \text{ control cells}}$$

Each point represents the average and standard error resulting from at least two independent experiments run in duplicate.

### Flow cytometry

The effect of a 2-h exposure to PYRZ on the cycle of OVCAR-3 cells was evaluated after recovery times of 6, 12 and 24 h. The cells were harvested by trypsinization at the appropriate times. After fixation in ethanol (70%, v/v), the cells were stained with an aqueous propidium iodide (PI) solution (100 µl, 100 µg $ml^{-1}$) containing RNAase (100 µl, 50 µg $ml^{-1}$) for 30 min at room temperature in the dark. The fluorescence was detected in a spectral range between 580 and 750 nm. Each cytometric analysis was performed on a Becton Dickinson FACScan instrument on 1–3 × $10^5$ cells. The percentage of cells in each cell cycle phase was estimated using LYSYS II software (Becton Dickinson).

### Transport study

Studies on kinetics of precursor transport were performed as described previously (Hayward et al, 1984). The OVCAR-3 cells were seeded for 24 h in 24-well plates at a density of 6 × $10^5$ cells per well. PYRZ was added to the medium at various concentrations. After a 2-h treatment the drug was removed by washing with phosphate-buffered saline (PBS) (2 × 2 ml) and fresh medium was added. The cells were allowed to recover for 3 h, after which fresh medium containing 0.3 µCi $ml^{-1}$ [$^{14}$C]thymidine was added. Label uptake was allowed to proceed for various intervals at 0°C (2, 5, 10, 20 or 35 min). The medium was removed and the wells rapidly washed with cold PBS (2 × 2 ml). The cells were detached with 100 µl of

© Cancer Research Campaign 1997

**Figure 7** DNA damage induced by PYRZ in OVCAR-3 cells. (-◆-) Internal control $^{14}C$-labelled DNA from untreated cells; (-△-) [$^{3}H$]thymidine DNA from treated cells. (A) 0 μM; (B) 50 μM; (C) 100 μM; (D) 200 μM

trypsin and dissolved in 3 ml of liquid scintillation fluid (Universol). The radioactivity was counted in a beta-scintillation counter.

### Sequence specificity of guanine-N-7 alkylation

Plasmid pRGM21, which was used to determine guanine specific N-7 alkylation, has been described in detail (Nobile et al, 1986). Briefly, the wild-type SV40 origin of replication from the *Hin*dIII to *Sph*I site (200 bp) was inserted between the *Hin*dIII and *Sph*I sites of pBR322. Using PCR, the SV40 region of the plasmid was amplified with the following primers: 5′-GGCCATCCAGCCTCG-3 and 5′-GTATCACGAGGCCCT-3′. The latter primer was end labelled (T4 polynucleotide kinase, Gibco BRL) with [$\gamma$-$^{32}$P]ATP (ICN Biomedical). The amplified labelled DNA (700 bp) was reacted with 1 mM PYRZ or temozolomide in 25 mM triethanolamine–1 mM EDTA, pH 7.2, at 37°C for 2 h. After precipitation and washing the DNA was treated with 1 M piperidine for 15 min at 90°C to produce breaks specifically at the sites of N-7 guanine alkylation. DNA fragments were separated on a 0.4-mm, 6% polyacrylamide gel in a solution containing 7 M urea and a Tris–boric acid–EDTA buffer system.

### Alkaline sucrose gradient

Cells were labelled for 24 h using a medium supplemented with 0.1 μCi ml$^{-1}$ [$^{3}$H]- or [$^{14}$C]thymidine for internal control cells (Zsido et al, 1991). Post-labelling (18–24 h) was performed before drug treatment. The drug was added for 2 h and the cells were allowed to grow in fresh medium for 24 h. They were then washed twice with PBS and dislodged by gentle scraping. To the $^{3}$H-labelled cells was added an aliquot of untreated $^{14}$C-labelled cells to serve as internal control during sedimentation analysis. The cells were then lysed in the dark at 0°C (lysis buffer: 0.55 N sodium hydroxide, 0.45 M sodium chloride, 10 mM $Na_2$ EDTA, 0.015% sarcosyl) and layered on the top of 5–30% sucrose gradients containing 0.3 N sodium hydroxide, 0.7 M sodium chloride, and 10 mM $Na_2$EDTA. Sedimentation was carried out at 4°C in a SW41 rotor of a centrifuge usually at 16 000 r.p.m. overnight. Gradients were fractioned by upward displacement with 1 ml of a dye dissolved in 40% sucrose and the samples collected with a BioRad fraction collector. The 0.3-ml fractions were analysed by single-phase liquid scintillation counting (dual-label settings).

### RESULTS

### Cytotoxicity

Survival curves for OVCAR-3 cells treated with PYRZ are shown in figure 2A and B. A clonogenic assay gave an $IC_{50}$ of 18 μM. The cell-killing kinetics, as determined by the sulphorhodamine B assay, showed that the rate of cell kill was rapid and time dependent only beyond a critical dose (around 20 μM). At high concentrations (50–100 μM), 90% kill was achieved within 24 h.

### Macromolecule synthesis

The effect of PYRZ on precursor incorporation was evaluated and the results are shown in Figure 3. Following a 2-h drug exposure and a 3-h recovery in drug-free media, a significant inhibition of DNA, RNA and protein synthesis was observed at high concentrations (50–100 μM). At 24 h recovery in drug-free medium, the inhibition of the synthesis of all three macromolecules was released. A slight increase in uridine and thymidine incorporation was apparent. Leucine uptake remained unchanged.

In order to determine whether the simultaneous inhibition of the synthesis of all three macromolecules was due to an alteration of precursor transports, the effect of PYRZ on the kinetics of label uptake was studied. As shown in Figure 4, no significant effect was observed on the rate of incorporation of thymidine at 3 h after treatment even at 100 μM, a concentration that induced a 50% inhibition of macromolecule synthesis.

### Flow cytometry

The effect of PYRZ on cell cycle was evaluated by flow cytometry (Figure 5). A dose-dependent increase in cell accumulation in late S and $G_2$+M was observed as early as 6 h after treatment. This effect was markedly enhanced at 12 and 24 h after treatment, with appearance at high concentrations of a sub-$G_1$ population of cellular debris at the left portion of the histograms. Two-dimensional forward light scatter DNA plots did not show any size reduction for these populations, thus excluding the possibility that they might be apoptotic cells.

### DNA damage

PYRZ (1 mM) was allowed to react for 2 h with the *Hin*dIII/*Sph*I sequence of SV40 DNA and the damage was analysed using the Maxam–Gilbert sequencing technique. Temozolomide induced significant levels of guanine N-7 alkylation. In contrast, PYRZ induced background levels of DNA alkylation (lane A) (Figure 6).

DNA damage induced by PYRZ in OVCAR-3 cells was studied by alkaline sucrose-density gradient as described (Zsido et al, 1991) (Figure 7). At 3 h post treatment, no DNA damage was observed over the whole dose range (data not shown). The DNA damage that appeared at 12 h at the high concentrations persisted at 24 h recovery and was accompanied by significant cell death.

### DISCUSSION

Our results clearly show that PYRZ is highly cytotoxic to OVCAR-3 cells. Cell-killing kinetics gave sigmoidal dose–response profiles, in contrast to those of temozolomide which, as we recently reported (Jean-Claude et al, 1996), showed a set of saturation curves. Thus, although PYRZ possesses the same structural elements as temozolomide (a 3-methyl-ureidotriazene portion), our data suggest that it is not behaving as a typical ureidotriazene-containing molecule. As shown in Figure 6, it exhibited much weaker interactions with a $^{32}$P-labelled DNA strand in vitro than did temozolomide at a supralethal concentration (1 mM). Surprisingly, despite inducing background levels of DNA alkylation, PYRZ was capable of causing significant DNA damage in OVCAR-3 cells at 10–20 times lower concentrations. This suggests that, in contrast to temozolomide, mechanisms other than direct DNA alkylation may contribute to the cytotoxicity of PYRZ.

PYRZ induced an unusual pattern of dose–response profiles for inhibition of macromolecule synthesis in OVCAR-3 cells. In contrast to mitozolomide, temozolomide, or BCNU (Horgan et al, 1984; Jean-Claude et al, 1996), which are known to selectively inhibit DNA synthesis, PYRZ showed a near-sigmoidal dose-inhibition profile with a plateau at 0–75 μM, and a dose-dependent inhibition of the synthesis of all three macromolecules (DNA, RNA and protein) at high concentrations. This inhibition is probably not

 © Cancer Research Campaign 1997

due to precursor transport, as we found that the rate of thymidine transport at 3 h after treatment was unaltered. At 24 h after treatment, no inhibition of macromolecule synthesis was apparent even at concentrations corresponding to 90% cell kill (50–100 μM). An increasing trend was apparent for RNA and protein synthesis. These effects were probably caused by the activation of several repair mechanisms that involved macromolecule synthesis. Base excision and long patch repairs of DNA, induction of transcription and de novo protein synthesis may be the cause of the apparent release of the inhibition of precursor incorporation. Moreover, the inhibition of macromolecule synthesis may have little implication in the mechanism of cell killing, as it occurred only at supratoxic concentrations.

The persistent DNA lesions induced by PYRZ were consistent with a progressive increase in cell cycle accumulation in late S and $G_2$+M in the 25–200 μM range. The block was reversed only at the lowest concentration (12.5 μM) 24 h after treatment. Cell cycle arrest in late S and $G_2$+M may be related to a delay in DNA repair processes (Hawn et al, 1995). This may cause the cells to miss essential transcripts required to progress through to the cycle and to die. At the concentrations that induced persistent cell cycle arrest and DNA damage, massive cell death was observed by both flow cytometric analysis and cell-killing kinetics.

Moreover, studies performed in our laboratory showed that temozolomide (**1**) an N-7- and O6-guanine alkylator (Catapano et al, 1987; Deans et al, 1992; Baer et al, 1993), induced delayed cell death and cell cycle arrest in OVCAR-3 cells. Other studies by Catapano et al (1987) showed that alkali-labile sites in L1210 cells treated with temozolomide (**1**) were repaired within about 24 h. This type of damage was therefore excluded as the lethal injury induced by temozolomide. It should also be noted here that temozolomide-treated OVCAR-3 cells arrested in late S and $G_2$+M, but were capable of escaping the block to later arrest in middle S, the next round of the cycle (Jean-Claude et al, 1996; Catapano et al, 1987). Cell death induced by temozolomide may be a delayed process highly related to point mutations induced by the O6-alkylguanine adduct. In contrast, PYRZ cytotoxic activity was rapid with maximal cell killing induced within one cell cycle time and, more importantly, cell cycle arrest induced by PYRZ was mostly irreversible and eventually led to cell death.

This study conclusively demonstrated that the novel heterocycle PYRZ, despite its structural similarities to temozolomide, may have a novel and different mechanism of cell killing.

### ACKNOWLEDGEMENTS

We thank the National Cancer Institute of Canada (NCIC) grant 4794 and the Luigi Barba Fund for financial support. We are also grateful to Dr Christopher Williams for theoretical calculations.

### REFERENCES

Baer JC, Freeman AA, Newlands ES, Watson AJ, Rafferty JA and Margison GP (1993) Depletion of O6-alkylguanine-DNA alkyltransferase correlates with potentiation of Temozolomide and CCNU toxicity in human tumour cells. *Br J Cancer* **66**: 1299–1302

Baig GU and Stevens MFG (1987) Antitumor imidazotetrazines. Part 12. Reactions of mitozolomide and its 3-alkyl congeners with oxygen, nitrogen, halogen, and carbon nucleophiles. *J Chem Soc Perkin Trans 1*, 665–667

Catapano CV, Broggini M, Erba E, Ponti M, Mariani L, Citti L and D'incalci M (1987) In vitro and in vivo methazolastone-induced DNA damage and repair in L-1210 leukemia sensitive and resistant to chloroethylnitrosoureas. *Cancer Res* **47**: 4884–4889

Chen J, Zhang Y, Moschel RC and Ikenaga M (1993) Depletion of O6-methylguanine-DNA methyltransferase and potentiation of 1,3-bis (2-chloroethyl)-1-nitrosourea antitumor activity by O6-benzylguanine in vitro. *Carcinogenesis* **14**: 1057–1060

Clark SA, Stevens MFG, Sansom CE and Schwalbe CH (1990) Antitumour imidazotetrazines, mitozolomide and Temozolomide. Probes for the major groove of DNA. *Anti-Cancer DrugDes* **6**: 63–68

Deans B and Tisdale MJ (1992) Antitumor imidazotetrazines XXVII: 3-methyladenine DNA glycosylase activity in cell lines sensitive and resistant to Temozolomide. *Cancer Lett* **63**: 151–157

Foedstad O, Aamdal S, Phil A and Boyd MR (1985) Activity of mitozolomide (NSC 353451), a new imidazotetrazine, against xenografts from human melanomas, sarcomas, and lung and colon carcinomas. *Cancer Res* **45**: 1778–1786

Gibson NW, Hartley JA, LaFrance RJ and Vaughan K (1986). Differential cytotoxicity and DNA-damaging effects produced in human cells of the Mer+ and Mer- phenotypes by a series of alkyltriazenylimidazoles. *Carcinogenesis* **2**: 259–265

Hamilton TA, Young TC, McKoy RC, Grotzinger WM, Green KR, Chu JA, Whang-Peng EW, Rogan J, Green AM and Ozols WR (1983) Characterization of a human ovarian carcinoma cell line (NIH: OVCAR-3) with androgen and estrogen receptors. *Cancer Res* **43**: 5379–5389

Hartley JA, Gibson NW, Kohn KW and Mattes WB (1986) DNA sequence selectivity of guanine-N7 alkylation by three antitumour chloroethylating agents. *Cancer Res* **46**: 1943–1947

Hawn MT, Umar A, Carethers JM, Marra G, Kunkel TA, Boland CR and Koi M (1995) Evidence for a connection between the mismatch repair system and the $G_2$ cell cycle checkpoint. *Cancer Res* **55**: 3721–3725

Horgan CM and Tisdale MJ (1984) Antitumour imidazotetrazines-IV. An investigation into the mechanism of antitumour activity of a novel and potent antitumour agent, mitozolomide. *Biochem Pharmacol* **33**: 2185–2192

Hayward IP and Parson PG (1984) Epigenetic effects of the methylating agent 5-(3-methyl-1-triazeno)-imidazole-4-carboxamide in human melanoma cells. *Austral J Exp Biol Med Sciences* **62**: 597–606

Jean-Claude BJ (1992) Synthesis and spectral studies of tetrazepinones. McGill University: Montreal

Jean-Claude BJ and Just G (1991) Synthesis of bi- and tri-cyclic tetrazepinones. *J Chem Soc Perkin Trans 1* 2525–2529

Jean-Claude BJ and Just G. (1997) Synthesis of pyridine fused tetrazepinones. *Heterocycles* (submitted)

Jean-Claude BJ and Williams CI (1997) $^{15}$N NMR of bi- and tricyclic tetrazepinones, *Magn Res Chem* (in press)

Jean-Claude BJ, Damian S, Damian Z, Do Khan L, Chan TH and Leyland-Jones B (1994) Tetrazepinones: a new class of DNA-directed antitumor agents. *Proc Am Assoc Cancer Res* **35**: 402

Jean-Claude BJ, Mustafa A, Damian Z, De Marte J, Chan Th and Leyland-Jones B (1995) The tetrazepinones are equally active in alkylating agent-resistant (Mer+) and sensitive (Mer–) human tumour cell lines. *Can J Infect Dis* **6** (suppl. C): 465C

Jean-Claude BJ, Mustafa A, Damian Z, De Marte J, Yen R, Chan TH and Leyland-Jones B (1996) Mechanism of 8-carbamoyl-3-methylimidazo [5,1-d]-1,2,3,5-tetrazin-4(3H)-one (Temozolomide)-induced cytotoxicity in the epithelial ovarian tumour cell line OVCAR-3: cell-cycle and cell-killing kinetics. *Biochem Pharm* (submitted)

Krishnan R, Binkley JS, Seeger R and Pople JA (1980) Self-consistent molecular orbital methods. XX. A basis set for correlated wave functions. *J Phys Chem* **72**: 650–655

Lowe PR, Sansom CE, Schwalbe CH, Stevens MFG and Clark AS (1992) Antitumour imidazotetrazines 25. Crusted structure of 8-carbamoyt-3-methyl-imidazo[5,1-d]-1, 2, 3, 5-tetrazin-4(3H)-one (Temozolomide) and structural comparisons with related drugs mitozolomide and DTIC. *J Med Chem* **35**: 3377–3382

Nobile C and Martin RG (1986) Stable stem-loop and cruciform DNA structures: isolation of mutants with rearrangements of the palindromic sequence at the simian virus 40 replication origin. *Intervirology* **25**: 158–171

O'Reilly SM, Newlands ES, Glaser MG, Brampton M, Rice-Edwards JM, Illingworth RD, Richards PG, Kennard C, Colquhoun IR, Lewis P and Stevens MFG (1993) Temozolomide: a new oral cytotoxic chemotherapeutic agent with promising activity against primary brain tumours. *Eur J Cancer* **29**: 940–942

Skehan P, Storeng R, Scudiero D, Monks A, McMahon J, Vistica D, Warren JT, Bokesch, Kenney HS and Boyd MR (1991) New colorimetric cytotoxicity assay for anti-cancer drug screening. *J Natl Cancer Inst* **82**: 1107–1012

Zsido TJ, Woynarowski JM, Baker RM, Gawron LS and Beerman TA (1991) Induction of heat-labile sites in DNA of mammalian cells by the antitumor alkylating drug CC-1065. *Biochemistry* **31**: 3733–3738

© Cancer Research Campaign 1997